# Comparative metabolomic profiling of tissue-specific Korean red pepper (*Capsicum annuum* L.) powders from Subicho and Bulcolor cultivars

**DOI:** 10.1007/s10068-026-02274-x

**Published:** 2026-09-03

**Authors:** Hahyeong Yu, Eunyoung Park, Ji-Ho Lee, Seoyeong Kim, Kyung-Hyung Ku, Jeong Hee Choi, Hyang Sook Chun, Jeong-Ho Lim, Jihyun Lee

**Affiliations:** 1 Department of Food Science and Technology, Chung-Ang University, Anseong, 17546 Republic of Korea; 2 Department of Food and Nutrition, Seoul National University, Seoul, 08826 Republic of Korea; 3 The Research Institute of Human Ecology, Seoul National University, Seoul, 08826 Republic of Korea; 4 Department of Biological Environment, School of Natural Resources and Environmental Science, Kangwon National University, Chuncheon, Gangwon State 24341 Republic of Korea; 5 Enterprise Solution Research Center, Korea Food Research Institute, Wanju, 55365 Republic of Korea; 6 Smart Manufacturing Research Group, Korea Food Research Institute, Wanju, 55365 Republic of Korea

**Keywords:** Metabolomics, Hot air-dried red pepper, Korean cultivars, Essential fatty acids, Essential amino acids

## Abstract

Hot air-dried red peppers are consumed as spices worldwide for their color, flavor, and nutritional value. However, comprehensive metabolomic studies are limited. This study addresses this research gap by investigating metabolite profiles of two Korean red pepper cultivars (Subicho, landrace; and Bulcolor, virus-resistant cultivar) across three different tissues and two harvest time points. Comprehensive profiling using GC–QqQ MS, HPLC–DAD, and UHPLC–MS platforms identified 160 primary metabolites and multiple secondary metabolite classes including capsaicinoids, carotenoids, and polyphenols. The placenta of Subicho contained markedly higher levels of essential amino acids, whereas Bulcolor exhibited elevated sterols, tocopherols, and essential fatty acids associated with virus resistance. Capsaicinoid content was highest in the placenta tissues, especially in the Subicho at early harvest (16,879.5 ± 398.3 μg/g), whereas the pericarp tissues were the primary sites for carotenoid accumulation. Principal component analysis resolved clear separation (PC1: 58.9%, PC2: 30.8%) by cultivar, tissue, and harvest timing. The high-resolution cultivar-, tissue-, and harvest-specific metabolomic database established in this study provides a critical scientific reference for hot air-dried red peppers. This resource offers a foundation for optimized cultivar selection, targeted utilization of specific tissues, and rigorous quality assessment in breeding programs, industrial production, and evidence-based consumer decision making.

**Supplementary Information:**

The online version contains supplementary material available at https://doi.org/10.1007/s10068-026-02274-x.

## Introduction

Peppers (*Capsicum annuum* L.) are globally consumed for their distinctive color, flavor, and nutritional properties. They are characterized by bioactive compounds, including capsaicinoids, which provide their pungency, along with carotenoids, polyphenols, and essential metabolites, which contribute to their flavor profile and health benefits (Guclu et al., 2021). Among the many functional compounds contained in peppers, it has been reported that the placenta is rich in capsaicin and flavonoids, whereas the pericarp contains a variety of glycosylated compounds (Cervantes-Hernández et al., 2019)

Peppers are consumed in multiple forms (e.g., fresh, dried, powdered, and paste), with powder being a significant consumption form in Korea (Lee et al., 2011). Peppers are dried for processing and preservation due to their high moisture content and perishable structure. Therefore, the determination of the properties of powders made from dried peppers is important for the functional and commercial considerations of powdered products.

However, most metabolite profiling studies have focused on fresh or freeze-dried samples (Cervantes-Hernández et al., 2019; Fei et al., 2022; Lozada et al., 2023; Wahyuni et al., 2013), leaving a knowledge gap regarding the biochemical characteristics of hot air-dried peppers. Additionally, previous research has typically examined specific metabolite groups related to pungency, color, and antioxidant properties (Korkmaz et al., 2021; Lahbib et al., 2017), providing a limited understanding of how cultivar, tissue type, and harvest timing simultaneously influence comprehensive metabolite profiles.

To address these limitations, this study investigated metabolite profiles across different fruit tissues (pericarp, placenta, and seed) of two Korean red pepper cultivars, Subicho and Bulcolor, at two harvest times. Subicho is a traditional Korean landrace (Sebastin et al., 2015), whereas Bulcolor is a disease-resistant cultivar bred against the resistance-breaking tomato spotted wilt virus (TSWV-WJ) isolate (Kwon et al., 2021). Evaluation of these cultivars enabled characterization of cultivar-dependent variation in metabolites associated with red pepper powder quality, including pungency, color, and nutritional attributes.

The aim was to construct a comprehensive, high-resolution metabolomic database for hot air-dried Korean red peppers, including primary metabolites (such as amino acids, lipid acids, and sugars), as well as capsaicinoids, carotenoids, and flavonoids. This dataset extends beyond traditional quality markers, providing a valuable resource for breeding and selection strategies aimed at enhancing nutritional and functional attributes in capsicum peppers.

## Materials and methods

### Chemicals, reagents, and standards

Capsaicinoid standards (capsaicin, 98.5%; dihydrocapsaicin, 97%) were purchased from Sigma-Aldrich (St. Louis, MO, USA). Carotenoid standards (capsanthin, 95%; α-carotene, 98%; β-carotene, 95%; β-cryptoxanthin, 97%; violaxanthin, 95%; and zeaxanthin, 95%) were purchased from Extrasynthese (Genay Cedex, France) or Sigma-Aldrich. Phenolic compound standards (3,4-dihydroxy benzoic acid, 97%; 4-hydroxybenzoic acid, 99%; apigenin, 95%; caffeic acid, 98%; chlorogenic acid, 95%; *p*-coumaric acid, 98%; gallic acid, 99%; luteolin-7-*O*-glucoside, 98%; naringenin, 98%; naringenin-7-*O*-glucoside, 95%; quercetin-3-*O*-glucoside, 98%; quercetin 3-*O*-rhamnoside, 95%; rutin, 95%; salicylic acid, 98%; sinapic acid, 98%; *t*-ferulic acid, 99%; and vanillic acid, 97%) were purchased from Sigma-Aldrich. Among the phenolic compound standards, quercetin-3,4'-*O*-diglucoside, 98.5% was obtained from Extrasynthese. Adonitol, 99%, GC derivatization grade methoxyamine hydrochloride (MeOX), and butylhydroxytoluene (BHT) were purchased from Sigma-Aldrich.

HPLC-grade formic acid, butylhydroxytoluene, and pyridine were purchased from Sigma-Aldrich. Acetonitrile (ACN) and methanol (MeOH) were purchased from J.T. Baker (Radnor, PA, USA) and Honeywell Burdick & Jackson (Morristown, NJ, USA), respectively. *N*-Methyl-*N*-(trimethylsilyl)trifluoroacetamide with 1% trimethylchlorosilane (MSTFA with 1% TMCS), hexane, and methyl tert-butyl ether (MTBE) were obtained from Thermo Fisher Scientific Co. (Asheville, NC, USA). Nanopure water was obtained from a water purification system (Milli-Q Direct 8, Merck Millipore, Billerica, MA, USA). The polyvinylidene fluoride (PVDF) and nylon syringe filters were purchased from Whatman (Maidstone, UK). The polytetrafluoroethylene (PTFE) syringe filter was purchased from Sartorius (Göttingen, Germany).

### Dried red pepper samples

Two red pepper cultivars (Subicho and Bulcolor) were harvested in 2021 from the Yeongyang Pepper Research Institute, Rural Development Administration (RDA), Republic of Korea. The Subicho and Bulcolor peppers were harvested twice on August 5 and September 6. The harvested red pepper fruits were hot air-dried by moisture drying at 65 °C for 7 h, followed by blow drying at 55 °C for 12 h and at 50 °C for 35 h. The three pepper tissues (pericarp, placenta, and seed) were separated using a scalpel. For analysis of the dried red pepper tissues, samples were ground using a grinder (SNSG-1002SS, Hanil Electronic INC., Seoul, Korea) and passed through a 30-mesh standard sieve. The powdered hot air-dried samples were immediately frozen and stored at -80 °C until analysis. Prior to chemical analysis, the powdered samples were further lyophilized to remove residual moisture, and all metabolite contents were expressed on a dry weight basis.

For each sample group, five independently prepared extracts were used for GC–MS/MS-based primary metabolite profiling (n = 5), whereas three independently prepared extracts were used for capsaicinoids, carotenoid, and phenolic compound analyses (n = 3).

### Profiling of primary metabolites in dried red pepper samples by GC–MS/MS

Primary metabolite profiling was performed using GC–MS/MS after methoximation and silylation derivatization as a targeted MRM-based approach. A 20 mg sample was weighed in a 2 mL Eppendorf tube (n = 5 per sample group). Then, the samples were extracted by sonication in a methanol solution containing 0.2 µg/mL of ribitol at a concentration of 20 mg/mL for 10 min at room temperature. The extracted samples were shaken for 1 h using a shaker (BS-31, JEIO TECH, Daejeon, Korea). Following this, centrifugation was conducted at 15,928 × *g* for 10 min using a Labogene instrument from Denmark. Using a speed vacuum concentrator (SPD2030, Thermo Fisher Scientific), 100 µL of the supernatant from the extracts was dried. For gas chromatography-triple quadrupole mass spectrometry (GC–MS/MS) analysis, methyloxime derivatives were obtained by dissolving the dried extracts in 50 µL of methoxyamine-HCl (20 mg/mL in pyridine) at 37 °C for 90 min. After methoximation, silylation was performed at 37 °C for 30 min by adding 50 µL of N-methyl-N-(trimethylsilyl) trifluoroacetamide (MSTFA) and 1% trimethylchlorosilane (TMCS).

The metabolites were analyzed using a Shimadzu GCMS-TQ8050 instrument in multiple reaction monitoring (MRM) acquisition mode. A total of 394 metabolites were determined, consisting of 332 metabolites from the Smart Metabolites Database (Shimadzu) and 62 metabolites from an in-house library. The injection volume was 1.0 µL, and two different split ratios (20:1 and 600:1) were applied due to differences in metabolome sensitivity. Separation of metabolites was achieved using a BPX-5 column (30 m × 0.25 mm i.d., 0.25 µm film thickness; TRAJAN Scientific and Medical, Ringwood, Australia). The temperatures of the injector, ion source, and transfer line were set to 250 °C, 230 °C, and 280 °C, respectively. The initial oven temperature was 60 °C (held for 2 min), then ramped to 320 °C (at a rate of 10 °C/min), and held for 15 min. Helium was used as the carrier gas with a flow rate of 1 mL/min, and argon was used as the collision gas. The electron ionization energy was set at 70 eV.

Shimadzu's GCMSsolution software (version 4.3) was used to process the data. Peak integration was assessed manually based on retention time consistency, MRM transition from the Smart Metabolites Database or in-house library, peak shape, and the absence of interfering signals. The peak areas of the metabolites and the internal standard ribitol were compared to determine the relative area of each individual metabolite.

### Determination of capsaicinoid in dried red pepper samples by HPLC

Sample preparation for capsaicinoid analysis in the pericarp, placenta, and seed of dried red pepper cultivars was performed using a previously described method with some modifications (Karnka et al., 2002), considering the limited amount of placenta. Sixty milligrams of seed, placenta, and pericarp were precisely weighed. Then 1 mL of acetonitrile was added, and the mixture was mixed well using a Genie 2 vortex (Scientific Industries, Bohemia, NY, USA). After that, extraction was performed at 40 °C for 30 min using an ultrasonic bath (Barsonic CPX3800H-E, Emerson, Danbury, CT, USA), followed by centrifugation at 13,000 rpm for 5 min. The supernatant was filtered through a 0.2-µm PTFE syringe filter.

Capsaicinoids in dried red pepper pericarp, placenta, and seed samples (n = 3 per sample group) were determined using an Agilent 1260 Infinity II HPLC system (Agilent Technologies, Santa Clara, CA, USA) with a UV detector. The column used was an XTerra^TM^RP18 column (4.6 × 150 mm, 5 µm, Waters, Milford, MA, USA) maintained at 35 °C. The mobile phase consisted of a gradient of 1% acetic acid in water (A) and acetonitrile (B) as follows: 40% B, 0–4 min; 40–62% B, 4–15 min; 62–80% B, 15–16 min; 80% B, 16–20 min. The flow rate was 1 mL/min, detection was at 280 nm, and the injection volume was 20 µL. Capsaicin and dihydrocapsaicin standards were dissolved in methanol and diluted to concentrations of 2.5, 5, 10, 30, 60, and 120 µg/mL for the calibration curve and quantified.

### Determination of the carotenoids in dried red pepper samples by HPLC–DAD

The carotenoid content was determined by analyzing the pericarp, placenta, and seed of the dried red pepper samples (n = 3 per sample group) using a modification of the method described in a previous study (Bonaccorsi et al., 2016). Lyophilized powder of the hot air-dried red pepper sample (100 mg) was extracted with 1 mL of hexane containing 0.1% butylhydroxytoluene and 1 mL of ethyl acetate containing 0.1% butylhydroxytoluene. The mixture was sonicated for 15 min in a dark room and then centrifuged for 15 min at 3,000 × *g* at 4 °C. Extraction was repeated until the residue was colorless. The collected supernatant was concentrated using nitrogen gas. The extracts were resuspended in 1 mL of MTBE/methanol (1:1 v/v) and filtered through a 0.2-µm nylon syringe filter.

The carotenoid content was quantified using an Agilent 1260 Infinity II HPLC system (Agilent Technologies) with a diode array detector (DAD). The carotenoid compounds were separated on a YMC C_30_ column (4.6 × 250 mm i.d.) packed with 1.9 µm particles (YMC Europe, Dinslaken, Germany). The mobile phases were MeOH/MTBE/water (81:15:4, v/v/v; solvent A) and MeOH/MTBE/water (7:90:3, v/v/v; solvent B), and the following gradient: 0–60% B, 0–54 min; 60–100% B, 54–65 min. The injection volume was 10 µL, and the column oven temperature was maintained at 25 °C. The flow rate was 1.0 mL/min. The detection was performed at the following wavelengths: 440 nm for violaxanthin and zeaxanthin; 450 nm for α-carotene, β-carotene, and β-cryptoxanthin; 470 nm for capsanthin. For the calibration curve, the carotenoid standards were dissolved in MTBE/methanol (1:1, v/v) and diluted to concentrations of 0.5, 1, 2.5, 5, 10, 25, 50, and 100 µg/mL. The limit of quantitation (LOQ) for each carotenoid was determined based on a signal-to-noise ratio of 10.

### Profiling of phenolic compounds in dried red pepper samples by UHPLC-Q-Orbitrap MS and UHPLC-QqQ-MS

Phenolic compounds were analyzed using a two-step workflow that integrated UHPLC-Q-Orbitrap MS-based annotation, based on accurate mass and MS/MS fragmentation data, with UHPLC-QqQ-MS-based MRM quantification. The phenolic compounds in the dried red pepper samples (n = 3 per sample group) were determined using a modification of a method from a previous study (Morales-Soto et al., 2013). The pepper seeds were defatted using a previously described method (Rahman et al., 2018), with some modifications. The red pepper seeds were vortexed with hexane (1:5, w/v) for 5 min 3 times using a Genie 2 vortex (Scientific Industries). The defatted pepper seeds were filtered through a Whatman filter paper and placed at room temperature overnight to allow the residual hexane to evaporate. Defatted seed samples were immediately used for phenolic extraction.

Approximately 0.15 g of lyophilized hot air-dried red pepper powder was weighed, and 5 mL of 80% aqueous methanol was added. The homogenate was sonicated for 15 min at 4 °C and then centrifuged at 10,621 × *g* at 5 °C for 10 min. The supernatants were collected and concentrated using a speed vacuum concentrator (SPD2030, Thermo Fisher Scientific). Finally, the concentrate was resuspended in 1 mL of 0.1% formic acid in water. The extracts were filtered through a 0.2-µm PVDF filter (Whatman), and the filtrates were used for UHPLC-Q-Orbitrap MS and UHPLC-QqQ-MS analyses.

For qualitative annotation, phenolic compounds in the pericarp, placenta, and seed of first-harvest Subicho samples were analyzed using an Ultimate 3000 UHPLC system coupled to a Q-Exactive Orbitrap high-resolution mass spectrometer (Thermo Fisher Scientific). Phenolic compounds were separated using a Poroshell C18 column (2.1 mm × 100 mm, 2.7 µm; Agilent Technologies) at 30 °C. The mobile phase consisted of a gradient of 0.1% aqueous formic acid (A) and 0.1% formic acid in acetonitrile (B) as follows: 5–10% B, 0–5 min; 10–12% B, 5–8 min; 12–15% B, 8–10 min; 15–15% B, 10–15 min; 15–55% B, 15–18 min; 55–90% B, 18–20 min; 5% B, 20–24 min. A negative ESI mode was used. The flow rate was 0.4 mL/min, and the injection volume was 5 µL. The MS data were obtained in full scan mode and data-dependent MS^2^ mode. The scan range was 80–1,000 m*/z*. The resolution of the MS scan was 70,000, and the resolution of MS^2^ was 17,500. The applied ionization parameters were set as follows: spray voltage at 2.5 kV, capillary temperature at 370 °C. The sheath gas flow rate, auxiliary gas flow rate, and S-lens RF level were set to 60, 20, and 55 a.u., respectively.

For targeted quantification, phenolic compounds selected based on the UHPLC-Q-Orbitrap MS data were analyzed using a 1290 ultra-high-performance liquid chromatograph (Agilent Technologies) coupled with a 6470 LC/TQ triple quadrupole mass spectrometer (Agilent Technologies). Phenolic compounds were separated using a Poroshell C18 column (2.1 mm × 100 mm, 2.7 µm; Agilent Technologies) maintained at room temperature. The mobile phase consisted of a gradient of 0.1% formic acid in water (A) and 0.1% formic acid in acetonitrile (B) as follows: 5–10% B, 0–5 min; 10–12% B, 5–8 min; 12–15% B, 8–10 min; 15–15% B, 10–15 min; 15–55% B, 15–18 min; 55–90% B, 18–20 min. The flow rate was 0.4 mL/min, and the injection volume was 5 µL. A negative ESI mode was used. The drying gas temperature and flow rate were 225 °C and 8.0 L/min, respectively. The sheath gas temperature and flow rate were 300 °C and 10 L/min, respectively. The nebulizer gas pressure, capillary voltage, and dwell time were 45 psi, 3.5 kV, and 5 ms, respectively. The MRM mode was used.

### Statistical analysis

Statistical analyses were conducted using IBM SPSS Statistics software (v. 23.0, SPSS, Inc., Chicago, IL, USA). Significant differences in the content of capsaicinoids, carotenoids, and phenolic compounds among the samples were confirmed by an analysis of variance (ANOVA).

Hierarchical cluster analysis and visualization of differentially expressed metabolites were performed using MetaboAnalyst 6.0 (www.metaboanalyst.ca). Hierarchical cluster analysis was employed to visualize the grouping of samples according to cultivar, tissue type, and harvest time, and the results were presented as heatmaps. Volcano plots were generated to identify differential metabolites between cultivars; for these analyses, mean values from two harvest times were computed for each tissue type of the two cultivars. Principal component analysis (PCA) biplots were constructed to explore overall variation and clustering patterns across all groups.

## Results and discussion

### Primary metabolites in dried red pepper

**Fig. 1:**
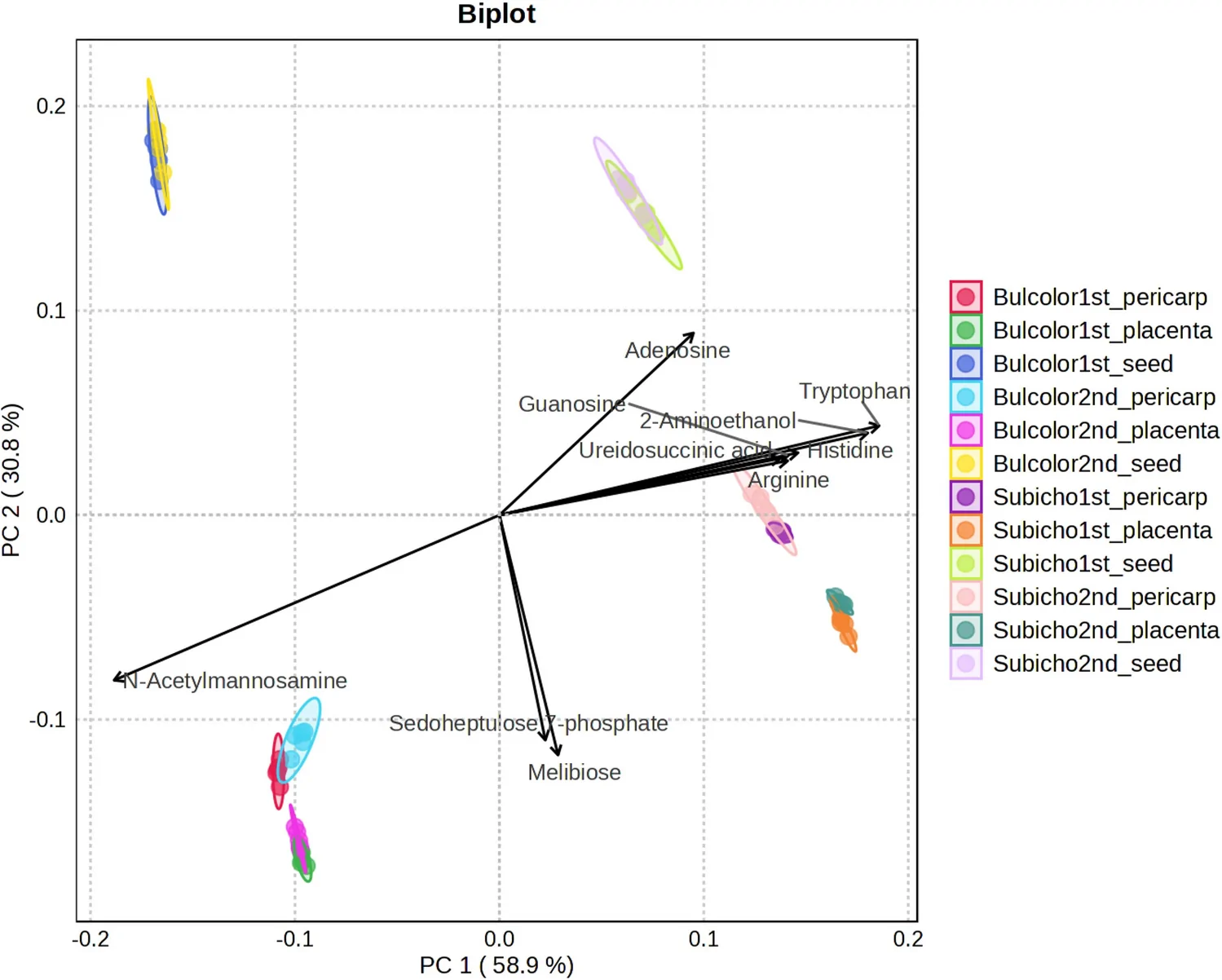
PCA biplot of 160 metabolites profiled in three fruit tissues (pericarp, placenta, and seed) from two red pepper cultivars (Subicho and Bulcolor) harvested at two different time points (*n* = 5)

**Fig. 2:**
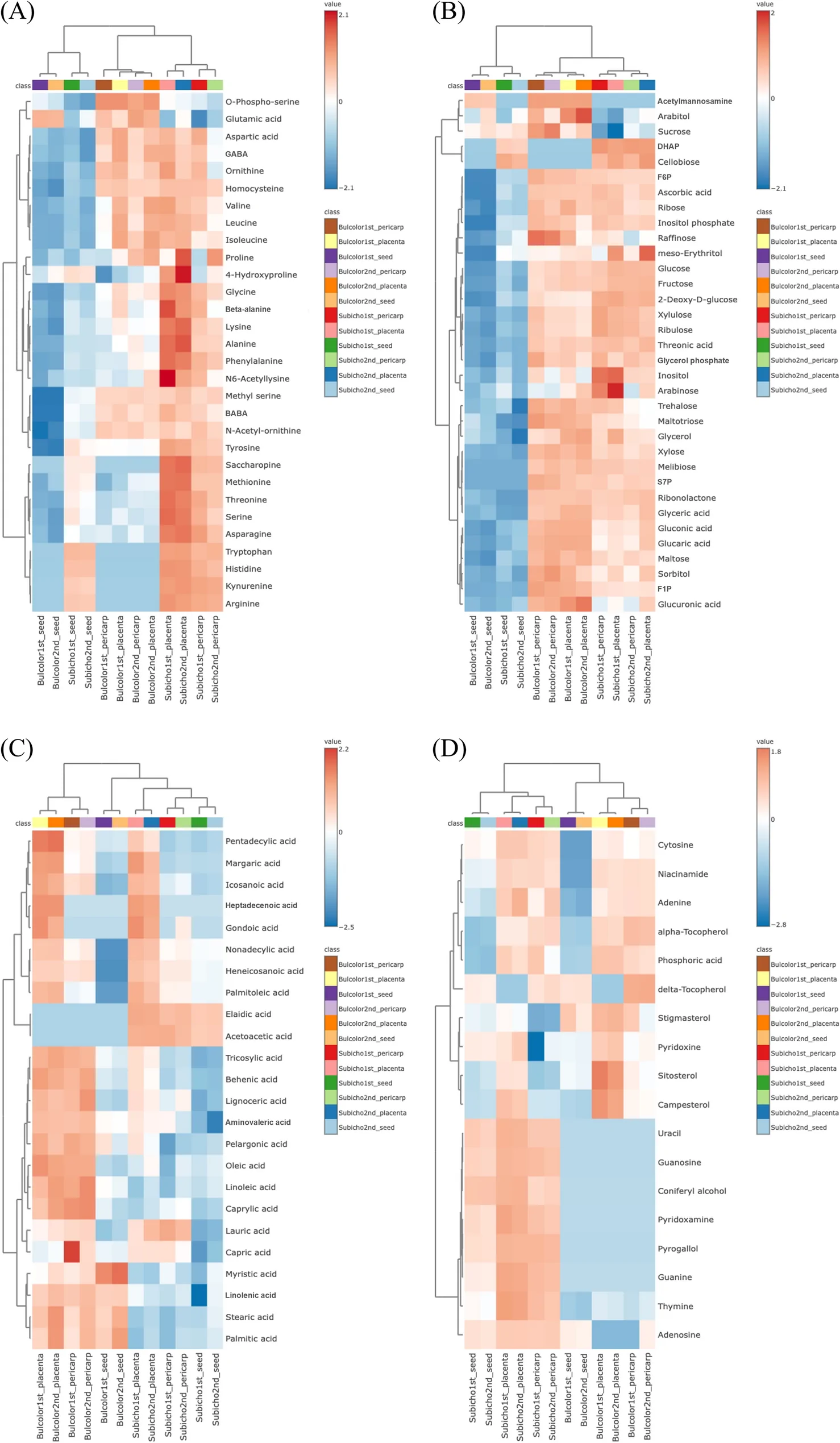
Heatmap of metabolite profiles showing clustering across fruit tissues, harvest times, and cultivars. **A** amino acids, **B** sugars, sugar alcohols, and sugar acids, **C** fatty acids, and **D** functional metabolites (*n* = 5)

**Fig. 3:**
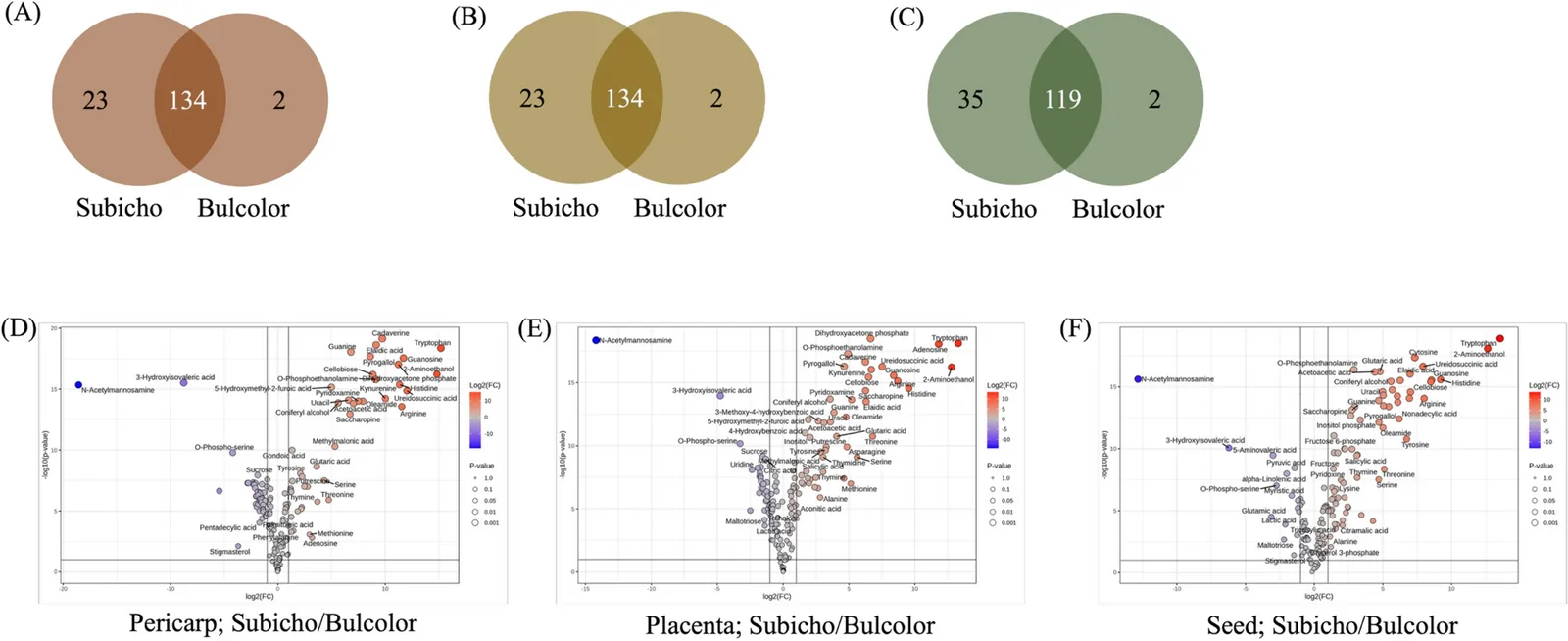
Comparative analysis of pepper cultivars. Venn diagrams show shared and unique metabolites in **A** pericarp, **B** placenta, and **C** seed. Volcano plots describe differential metabolite abundance between Subicho and Bulcolor in **D** pericarp, **E** placenta, and **F** seed. Data represent the mean of two harvest points (*n* = 5). * Volcano plots are graphical representations that display the fold change (log_2_(FC)) versus statistical significance (-log_10_(*p*-value)) of the metabolomic data

One hundred and sixty primary metabolites were identified and quantified in pepper samples using GC-QqQ MS, as shown in Supplementary Table S1. Among the identified metabolites, 75 compounds were consistent with previously reported *Capsicum* metabolites, whereas 85 were either newly detected or remained structurally uncharacterized (Supplementary Table S1). To investigate patterns of metabolite variation, principal component analysis (PCA) was performed (Fig. 1). The two components explained 89.7% of the total variance, separating samples by cultivar (PC1, 58.9%) and tissue type (PC2, 30.8%). Similarly, hierarchical clustering based on heatmap analysis (Fig. 2) also indicated separation according to tissue and cultivar, whereas sample clustering was not significantly impacted by harvest timing. These results suggest that tissue- and cultivar-specific metabolic compounds are major factors in the observed variance. Given the limited impact of harvest timing on the overall clustering patterns, the first and second harvest values were averaged only for the Fig. 3 analyses, including Venn diagrams (Figs. 3A–C) and volcano plots (Figs. 3D–F) analyses. This approach enhanced the detection of meaningful differences across tissues and cultivars.

The targeted profiling of 30 amino acids exhibited cultivar and tissue specificity, with the Subicho placenta showing dominant enrichment of essential amino acids (e.g., threonine, methionine, tryptophan, histidine, lysine, and leucine) across all tissues analyzed (Fig. 2A and Figs. 3D–F). This profile suggests a higher capacity for protein biosynthesis in the Subicho reproductive tissue, consistent with the physiological role of the placenta as a metabolically active organ (Wang et al., 2018). Additionally, substantial accumulation of 4-hydroxyproline in the Subicho placenta suggests its contribution to the synthesis of hydroxyproline-rich glycoproteins, which are components of plant cell wall structural proteins (Cassab, 1998). Conversely, the Bulcolor exhibited comparatively higher levels of glutamic acid, a non-essential amino acid known for its umami flavor properties and its contribution to sensory quality in foods (Ardö, 2006). This suggests that while the Subicho placenta emphasizes biosynthetic function, the Bulcolor may favor flavor-enhancing amino acid accumulation.

The profiling of 34 sugars, sugar alcohols, and sugar acids showed cultivar- and tissue-specific patterns that correlate with sweetness characteristics (Fig. 2B). Subicho consistently showed higher concentrations of reducing sugars, such as glucose and fructose, specifically in both placenta and pericarp tissues, with levels approximately 1.5 to 1.7 times greater than those in the Bulcolor. Conversely, sucrose levels were notably higher in the pericarp of Bulcolor, where they were 3.7 times greater than those in Subicho. These patterns were supported by volcano plot analysis, which identified sucrose as significantly enriched in Bulcolor (Figs. 3D and 3E), and by heatmap clustering (Fig. 2B), which also separated samples by cultivar. These findings may provide a physiological basis for sensory variation and offer valuable guidance for breeding strategies aimed at flavor optimization in dried pepper products.

The composition of 24 fatty acids is shown in Fig. 2C. Bulcolor tissues showed higher concentrations of linoleic acid and α-linolenic acid, both of which are essential fatty acids in the human diet (Figs. 2C and 3F). These polyunsaturated fatty acids are also known as precursors of lipid-derived signaling compounds in plants. Previous studies have suggested that fatty acid desaturase 1 (CaFAD1) and 13-lipoxygenase (LOX) genes are associated with tobamovirus responses in *C. annuum*, suggesting a possible relationship between fatty acid metabolism and plant stress responses (Juhász et al., 2015; Kim et al., 2007). The comparatively higher levels of linoleic and linolenic acid observed in Bulcolor not only characterize its metabolic phenotype compared to that of the landrace cultivar (Subicho) but also provide biochemical evidence of its breeding background as a virus-resistant cultivar.

In addition to primary metabolites, vitamins, sterols, and nucleobases also contributed to the metabolic differences between the cultivars (Fig. 2D). The Bulcolor pericarp was enriched in α- and δ-tocopherol, compounds that function as antioxidants and may enhance protection against oxidative stress (Ali et al., 2022; Kuo et al., 2008). Similarly, stigmasterol and sitosterol were more abundant in Bulcolor, especially at the first harvest, which could contribute to membrane stability and resistance to environmental stress (Griebel and Zeier, 2010). In the Subicho placenta, higher levels of nucleobases such as thymine, cytosine, adenine, and guanine were detected, which may align with its abundance of amino acids (Minchin and Lodge, 2019).

Overall, comparative profiling suggested that Subicho was abundant in essential amino acids, highlighting its nutritional potential, whereas Bulcolor accumulated umami-related amino acids along with polyunsaturated fatty acids, tocopherols, and sterols, reflecting both sensory quality and defense metabolism. Tissue-specific partitioning further highlighted these differences, with sugars concentrated in the pericarp, a broad range of metabolites abundant in the placenta, and only a limited number of metabolites detected in seeds.

### Capsaicinoid content in dried red pepper

Quantitative analysis of capsaicin and dihydrocapsaicin showed tissue- and harvest-dependent variations in dried red pepper samples from the Subicho and Bulcolor cultivars (Table 1). The total capsaicinoid contents in pericarp, placenta, and seeds of the dried red pepper were 42.5–89.9, 8,148.1–16,879.5, and 17.3–85.7 µg/g, respectively. Among the analyzed tissues, the placenta showed the highest total capsaicinoid content. These findings align with previous reports indicating that although the placenta constitutes less than 4% of the total dry weight, it accounts for approximately 96% of the total capsaicinoid content (National Institute of Agricultural Science, 2012).

**Table 1:** Capsaicinoid content (µg/g) of different red dried pepper cultivars at different harvest times (*n* = 3)

	Capsaicin	Dihydrocapsaicin	Total capsaicinoid
Pericarp			
Subicho 1st	55.1 ± 2.9^e^	34.7 ± 2.9^e^	89.9 ± 5.7^e^
Subicho 2nd	24.4 ± 1.7^e^	18.0 ± 1.5^e^	42.5 ± 2.8^e^
Bulcolor 1st	36.9 ± 1.4^e^	17.6 ± 0.8^e^	54.5 ± 2.0^e^
Bulcolor 2nd	44.6 ± 3.2^e^	27.9 ± 0.2^e^	72.5 ± 3.4^e^
Placenta			
Subicho 1st	9,444.7 ± 211.2^a^	7,434.8 ± 188.0^a^	16,879.5 ± 398.3^a^
Subicho 2nd	5,091.8 ± 249.2^c^	4,533.8 ± 166.7^b^	9,625.6 ± 415.5^b^
Bulcolor 1st	5,514.9 ± 95.7^b^	3,599.4 ± 111.7^d^	9,114.4 ± 41.6^c^
Bulcolor 2nd	4,287.6 ± 128.9^d^	3,860.6 ± 118.6^c^	8,148.1 ± 246.4^d^
Seed			
Subicho 1st	50.8 ± 1.3^e^	34.9 ± 0.8^e^	85.7 ± 1.4^e^
Subicho 2nd	10.0 ± 0.5^e^	7.3 ± 0.3^e^	17.3 ± 0.6^e^
Bulcolor 1st	51.1 ± 0.9^e^	24.8 ± 0.8^e^	75.9 ± 1.3^e^
Bulcolor 2nd	19.0 ± 1.2^e^	13.2 ± 0.9^e^	32.2 ± 1.9^e^

Values are presented as mean ± SD (n = 3)

Mean values followed by different letters indicate significant sample differences at *p* < 0.05

In both cultivars, capsaicin concentrations were consistently higher in the first harvest compared to those in the second. For example, the Subicho placenta tissue contained 9,444.7 µg/g of capsaicin at the first harvest, which decreased to 5,091.8 µg/g at the second. Prior studies have shown that capsaicin levels can vary depending on external factors such as cultivation temperature, nutrient composition, and maturity (Rahman and Inden, 2012). Taken together with the previous study, the decline in capsaicin content at later harvests could be attributed to metabolic changes during ripening or enzymatic degradation. Specifically, the activity of peroxidase enzymes has been implicated in the post-maturation degradation of capsaicinoids (Vázquez-Espinosa et al., 2023).

These results show that the placenta was the principal site of capsaicinoid accumulation among the analyzed tissues, particularly in Subicho at the first harvest. Capsaicin concentrations were also higher at the first harvest than at the second harvest in both cultivars. These findings indicate that harvest timing can influence specific quality markers related to pungency, such as capsaicinoids, whereas its effect on the overall primary metabolite profiles was limited.

### Carotenoid content in dried red pepper samples

In this study, six carotenoids (violaxanthin, capsanthin, β-cryptoxanthin, zeaxanthin, α-carotene, β-carotene) were identified in the dried red pepper (Table 2), which represent the major previously reported carotenoid compositions (Arimboor et al., 2015). Carotenoids were detected in both the pericarp and placenta, but not in the seed. Among them, α-carotene was quantified only in the pericarp. Carotenoids were analyzed because they determine fruit coloration and are linked to biological functions. The carotenoids identified here have been associated with effects on gastric cancer, obesity, and tumor progression (Villa-Rivera and Ochoa-Alejo, 2020). In particular, capsanthin is the major carotenoid in peppers, and it has been reported to exhibit antioxidant and antitumor activities (Kim et al., 2009).

**Table 2:** Carotenoid content (µg/g) of different red pepper cultivars at different harvest times (*n* = 3)

	Violaxanthin	Capsanthin^*^	β-Cryptoxanthin	Zeaxanthin	α-Carotene	β-Carotene^*^	Total carotenoid
Pericarp							
Subicho 1st	22.1 ± 0.4^c^	179.2 ± 1.5^a^	9.6 ± 0.7^bc^	18.0 ± 0.2^b^	12.2 ± 0.8	55.0 ± 0.2^e^	296.0 ± 3.0^b^
Subicho 2nd	28.5 ± 0.2^b^	173.1 ± 2.3^a^	5.8 ± 0.1^e^	8.1 ± 0.1^d^	30.9 ± 2.4	49.0 ± 2.8^e^	295.4 ± 6.0^b^
Bulcolor 1st	29.9 ± 0.7^b^	179.9 ± 4.2^a^	9.5 ± 0.1^bc^	7.0 ± 0.3^de^	16.4 ± 1.3	75.0 ± 1.2^c^	317.6 ± 5.4^a^
Bulcolor 2nd	35.9 ± 1.5^a^	155.1 ± 6.1^b^	9.6 ± 0.3^bc^	5.9 ± 0.1 ^e^	27.0 ± 1.6	85.2 ± 3.3^b^	318.7 ± 12.3^a^
Placenta							
Subicho 1st	4.4 ± 0.2 ^f^	65.0 ± 1.8 cd	10.1 ± 0.1^ab^	35.1 ± 0.7^a^	< LOQ	67.3 ± 1.8^d^	181.9 ± 4.4^c^
Subicho 2nd	13.1 ± 0.5 ^d^	73.2 ± 2.2 ^c^	8.3 ± 0.4^d^	10.7 ± 0.5^c^	< LOQ	75.6 ± 2.3 ^c^	180.8 ± 5.4 ^c^
Bulcolor 1st	5.5 ± 0.2 ^f^	70.6 ± 3.6 ^c^	10.8 ± 0.4^a^	11.5 ± 0.9^c^	< LOQ	79.2 ± 2.9 ^bc^	177.6 ± 7.7 ^c^
Bulcolor 2nd	8.6 ± 0.6 ^e^	56.9 ± 2.5 ^d^	9.0 ± 0.5 cd	6.6 ± 0.2^e^	< LOQ	97.3 ± 4.0 ^a^	178.4 ± 6.8 ^c^
Seed							
Subicho 1st	< LOQ	< LOQ	< LOQ	< LOQ	< LOQ	< LOQ	< LOQ
Subicho 2nd	< LOQ	< LOQ	< LOQ	< LOQ	< LOQ	< LOQ	< LOQ
Bulcolor 1st	< LOQ	< LOQ	< LOQ	< LOQ	< LOQ	< LOQ	< LOQ
Bulcolor 2nd	< LOQ	< LOQ	< LOQ	< LOQ	< LOQ	< LOQ	< LOQ

Values are presented as mean ± SD (n = 3)

< LOQ, below the limit of quantitation. LOQ values were 0.04, 0.07, 0.10, 0.38, 0.32, and 0.20 µg/g for violaxanthin, capsanthin, β-cryptoxanthin, zeaxanthin, α-carotene, and β-carotene, respectively

Mean values followed by different letters indicate significant sample differences at *p* < 0.05

^*^Compounds with VIP value > 1

Multivariate analysis identified capsanthin and β-carotene as potential biomarker metabolites (Variable Importance in the Projection; VIP > 1 and *p*-value < 0.05), confirming their role as key contributors in differentiating tissues and cultivars. Capsanthin was consistently the predominant carotenoid, with levels ranging from 155.1 to 179.9 µg/g. Oxygenated carotenoids with acylcyclopentanol end groups, particularly capsanthin, are recognized as the main pigments responsible for red coloration in pepper fruits (Gómez-García and Ochoa-Alejo, 2013). Conversely, the placenta was characterized by β-carotene as the dominant carotenoid, with levels ranging from 67.3 to 97.3 µg/g. This distribution is consistent with previous findings that capsanthin and β-carotene are the major carotenoid compounds in red pepper (Wahyuni et al., 2011). Differences in carotenoid composition associated with harvest time were limited.

Altogether, total carotenoid content was consistently higher in the pericarp than that in the placenta. The Bulcolor contained higher levels than those in the pericarp of the Subicho. Capsanthin and β-carotene could be reliable markers of both color and functional quality in dried peppers.

### Polyphenol content in dried red pepper samples

Polyphenols, which contribute to antioxidant capacity and have been associated with biological activities such as anti-inflammatory and anticancer effects, were profiled to assess their nutritional and functional roles in peppers (Chel-Guerrero et al., 2021; Shahidi and Ambigaipalan, 2015). Thirty-nine phenolic compounds were identified in dried red pepper (Supplementary Table S2A). Of these, 17 were confirmed with standards and 22 were quantified (Supplementary Table S2B). The quantified compounds included one flavanone, three flavones, four flavonols, six hydroxybenzoic acids, and eight hydroxycinnamic acids.

**Fig. 4:**
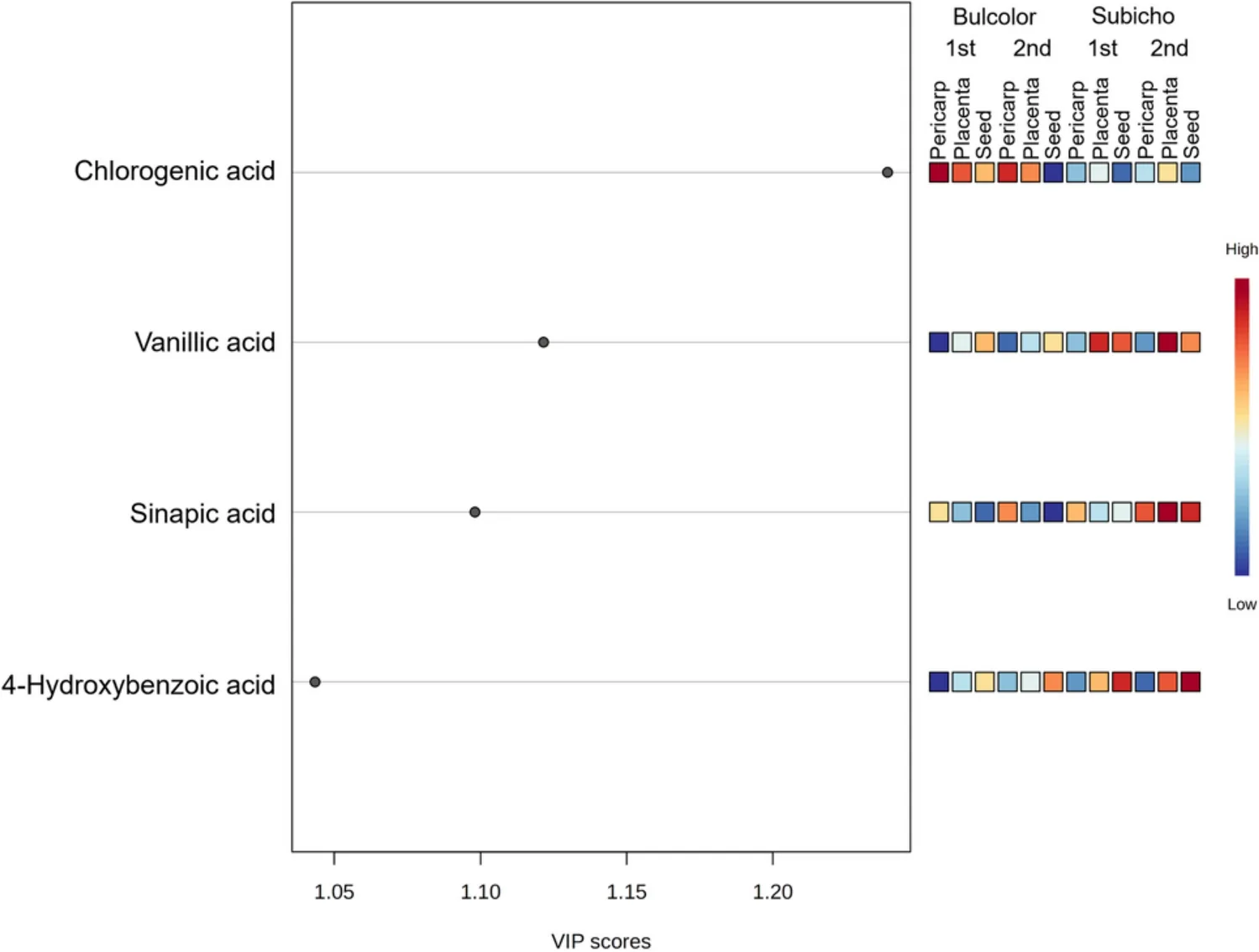
Variable importance in projection (VIP) scores for chlorogenic acid, vanillic acid, sinapic acid, and 4-hydroxybenzoic acid in the pericarp, placenta, and seed of two pepper cultivars (Bulcolor and Subicho) across two harvests. Data are shown for metabolites meeting the selection criteria (VIP > 1, *p*-value < 0.05), with heatmaps describing mean relative abundances (*n* = 3)

Phenolic composition varied mainly by tissue and cultivar, whereas harvest effects were limited and compound-specific. Multivariate analysis highlighted chlorogenic acid, vanillic acid, sinapic acid, and 4-hydroxybenzoic acid as the major discriminant metabolites differentiating cultivars, tissues, and harvests (VIP > 1 and *p*-value < 0.05) (Fig. 4). In the Bulcolor pericarp and placenta, chlorogenic acid was enriched and has been reported to have antioxidant and hepatoprotective effects (Ali et al., 2017; Yi et al., 2019). Vanillic acid, another potential marker, was abundant in the Subicho placenta and in the seeds of both cultivars, suggesting the role of hydroxybenzoic acids in reproductive tissues and supporting antioxidant and hepatoprotective activity (Arya et al., 2021; Avilés-Betanzos et al., 2023). Lastly, 4-hydroxybenzoic acid was concentrated in the seeds, providing antimicrobial and antioxidant capacity (Simal-Gandara and Prieto, 2020).

Collectively, these secondary metabolites demonstrate the relevance of these compounds for nutritional and functional properties and indicate how cultivar, tissue, and harvest timing specificity change phenolic distribution in peppers.

In conclusion, this study provides a metabolomic profile of hot air-dried Korean red peppers, showing that cultivar, tissue, and harvest time shape metabolite composition relevant to nutrition and functionality. Subicho, a traditional landrace, showed higher levels of essential amino acids and reducing sugars, supporting its nutritional quality. In contrast, Bulcolor, a virus-resistant cultivar, showed higher levels of glutamic acid, sucrose, and polyunsaturated fatty acids, suggesting metabolic differences associated with defense and flavor potential. The placenta was the major reservoir of capsaicinoids, the pericarp was enriched in sugars and carotenoids, and the seeds contained specific fatty acids and hydroxybenzoic acids. Capsaicin was strongly affected by harvest timing and peaked at the first harvest in both cultivars. Although cultivar and harvest coverage was limited, developing a comprehensive metabolite database could facilitate the strategic selection of pepper cultivars for specific purposes, such as enhanced flavor, spiciness, or health benefits.

## Supplementary Information

Below is the link to the electronic supplementary material.

Supplementary file1 (XLSX 165 KB)
